# Application of Chemistry to Practical Medicine

**Published:** 1850-07

**Authors:** M. M. Rogers

**Affiliations:** Author of Agricultural Chemistry,” “Scientific Agriculture,” &c. Rochester


ART. II.—*Application of Chemistry to Practical Medicine,* By M. M. Rogers, M. D., Author of Agricultural Chemistry," "Scientific Agriculture," &c.

The science of Chemistry has for its object the investigation of the properties of all elementary and compound substances, their relations and combinations, the agencies by which their changes are effected, and the laws which govern them. The basis on which this science rests, is facts and experiment; it is purely a demonstrative science, and no hypothetical or speculative views can be *practically* of any service in its advancement and application. Every change which takes place in the elementary constitution of matter in the universe, whether effected by natural causes, or by the operations of art, involves a fixed chemical law, and is due to chemical action.

Chemistry consists of two distinct branches, viz., analysis and synthesis.

Analysis consists in decomposing a compound body and separating its elements. Synthesis consists in uniting simple bodies so as to form a compound. The forces which preside over and cause chemical action are light, caloric, electricity, and magnetism; the relative importance of these forces cannot be exactly estimated in the present state of the science; the question of their individual distinctness or identity with electricity remains unsettled. The science of chemistry, which has achieved greater triumphs over matter, and conferred more practical knowledge of nature upon civilized man, than all other sciences combined, has gradually grown out of the superstitious art of alchemy. Modern chemistry, instead of alluring its votaries into a fruitless search after the "philosopher's stone," the "universal solvent," or the "universal catholicon," crowns their investigations with results which tend to the advancement of civilization, and the increase of human comforts and happiness. Its objects are not limited to the study of abstract laws alone, but extend also to the improvement of the useful arts, the cure of disease, the preparation of food, the developement of the laws of organic life, and, finally, to every thing affecting our physical relations to the material universe. No art or profession is so intimately connected with, and dependent upon chemistry, as that of the practice of medicine.

The elementary branches, Anatomy, Physiology, Botany, and Materia Medica, are so dependent upon chemistry for the evolution of their principles, that they were never understood, or investigated to much extent, until this science was made a basis of all philosophical research, in relation to the properties of matter. And the successful operations of the deductive branches, viz., Pharmacy and Therapeutics, and in some degree, Surgery and Obstetrics, bear an exact proportion to the amount of correct chemical knowledge employed in their prosecution. All success in the practical branches of medicine, is owing to the discovery and carrying out of chemical laws, although the practitioner may be ignorant of this fact, and may not recognise them as such.

If we admit that all changes whatever, which take place in the elementary constitution of matter, both organic and inorganic, are merely chemical transformations, we see that the practice of medicine proper, is only the aggregate of a series of chemical experiments, and the physician a practical chemist. When we reflect that, so far as yet known, we have but about sixty simple or uncombined elementary substances, and that from these every organic and inorganic compound is made up, we see that the number of chemical changes which take place between these few elements, is absolutely infinite. But a difference in elementary constituents and proportions, is by no means necessary in order to form different substances.

By a principle called *Isomerism*, several substances of very different properties are formed from the same elements, in exactly the same proportions. Cane sugar, milk sugar, and grape sugar, are each composed of carbon, hydrogen, and oxygen, each 12,—or equal parts of carbon and water. Woody fibre, starch, gum, and cane sugar, are each composed of carbon 12, hydrogen 10, and oxygen 10. Oil of turpentine and oil of citron are each composed of carbon 5, and hydrogen 4. Difference in internal molecular arrangement causes the difference in chemical and physical properties in isomeric compounds.

*Metamorphosis* of organic elements is another interesting point. There are certain organic compounds which, from the complexity of their constitution, and consequent weakness of affinity, are peculiarly disposed to decomposition and change of elementary form. They are disposed to change whenever the balance of opposing forces is destroyed: that is, whenever by the agency of some external disturbing force, as heat, air or water, the affinity which holds these elements together is overcome, the elements are separated entirely, or one element is replaced by another, and another compound formed. Lignine or wood fibre may be changed to starch by boiling in water, drying in an oven, and then fermenting with yeast. In this way the writer has made bread of green beech wood, which was but little inferior to that made from unbolted wheat flour. Woody fibre may be transformed to starch also, by the action of sulphuric acid or caustic potash. Starch when gradually heated to 300 deg. F. assumes a brownish tint, and is changed to gum. By the action of sulphuric acid, also, starch may be changed to gum, and this again to grape sugar. Gum arabic may be changed to sugar by the action of chalk and sulphuric acid. Cane sugar, when heated to 360 deg. F., gives off two atoms of water, and is changed to caramel. Cane sugar may be changed to grape sugar by digesting it in dilute sulphuric acid at a gentle heat. By the agency of casein, milk sugar is transformed to lactic acid, and this again to butyric acid.

*Animal chemistry* furnishes an explanation of almost every physiological and pathological change which takes place in animal bodies. A few brief remarks on the constitution of some of the animal fluids will show the agency of chemistry in the development of physiology and pathology.

There are four classes of diseases in which the *blood* undergoes peculiar changes. The *first* class includes acute inflammation, tubercle, and cancer. In these the quantity of fibrine in the blood is increased. The *second* class includes cerebral hemorrhages, and continued fevers without local inflammation. In this class the fibrine is diminished, and the globules increased in proportion to the fibrine. The *third* class includes chlorosis, marasmus, anemia, &c. In this class there is a great diminution of globules. In the *fourth* class, Bright's disease is an example. In this class the fibrine and globules are unchanged, but the quantity of albumen in the serum is diminished. Of all the constituents of the blood, the globules suffer the greatest changes. In acute inflammation there are three times as many globules as in chlorosis. Next to the globules, the fibrine suffers the greatest change in quantity. The salts are least liable to change in quantity, and vary the least in quality and relative proportions. The blood in cholera contains less fluid and alkaline salts, and more solid matter. In diabetes mellitus, the blood often contains traces of sugar. In jaundice the blood contains a small quantity of the green coloring matter of the *bile.* This matter is said to be identical with chlorophyde, the green coloring principle of plants. By the addition of nitric acid, bile assumes first a green, then blue, violet, red, and lastly yellow color. By different degrees of oxydation in this way, the various colored bile of different animals is formed. *Serum* contains two liquids, viz. albumen and pyen. They both coagulate by heat, acetic acid, and alum, so that this is no test for the distinction of pus. Pus globules always sink, while mucus swims in water; and this is the true test. The *liquor amnii* is said to be identical with dropsical fluid. *Chyle* and *lymph* are the same as blood, with an excess of water and he absence of hematosine. *Mucus* is dissolvod by alkalies and precipitated by acids and tannin. *Saliva* contains cyanogen in the form of a sulphocyanide of potassium. The urine, chemically considered, is of more importance to be well understood than any other fluid in the body. In its composition it is more complex than any other, and its affinities consequently weaker. It is also composed of those highly nitrogenized compounds which are peculiarly disposed to change of form. It being a fluid which is secreted and discharged from the body in large quantities there is no obstacle to chemical tests and inspection of its properties. From its complexity of constitution, its tendency to rapid decomposition, and its being the vehicle of several effete matters, we may reasonably look for more and greater alterations, both in health and disease, than in any other fluid. The substances which will be found under different circumstances, are urea, uric acid, albumen, coloring matter of the bile, phosphate of alkalies, lime, sulphates, hydro-chlorate, free sulphuric acid, and phosphoric acids, alkalies, fibrin, casein, mucus, blood, and sugar.

I may briefly mention some of the re-agents used in examining urine in disease. *Nitric acid* is used to detect urea, uric acid, albumen, and the coloring matter of the bile. *Lime water* shows the presence of alkaline phosphates, by precipitating phosphate of lime. *Ammonia* also precipitates phosphate of lime held in solution by free uric acid. *Oxalate of ammonia* precipitates carbonate of lime. *Ammonia* added to this, precipitates ammoniacal phosphate of magnesia. *Acetate of Baryta* shows the presence of sulphuric acid. *Neutral acetate of lead* precipitates the chloride and phosphate of lead,—these are distinguished by the flame given under the blowpipe. *Solution of alum* produces turbulence in urine that holds albumen or fibrine in solution. *Bichloride of mercury* gives a precipitate in acidified urine, if albumen or casein be present. *Tannin*, or infusion of *nut galls*, precipitates mucus and extractive matter; the latter is precipitated also by *acetate of lead*. *Red* and *litmus paper* are used as a general test for alkalies and acids. *Yeast*, by exciting vinous fermentation in urine, shows the presence of sugar. A substance called *keistine* is said to be an evidence of pregnancy, if found in the urine. It is a cream-like matter which collects on the surface of urine after standing for a few days in a glass vessel. This test has some exceptions.

The *tests* for several *metals*, whether found in the system or elsewhere, are important to be known. The test for silver, lead, copper, antimony, tin, and arsenic, is sulphuretted hydrogen. The test for gold is proto-sulphate of iron; for zinc, hydro-sulphate of ammonia; for iron, ferro-cyanide of potassium.

The *antidotes* for various poisons may be briefly enumerated. The antidote for poisoning by antimony, is tannin or nut galls, or yellow cinchona; copper, any form of albumen; arsenic, hydrated per-oxide of iron; corrosive sublimate, albumen; nitrate of silver, common salt; nitrate of potash, no chemical antidote; acetate of lead, epsom salts; sulphate of zinc, chromate of potassa, milk or albumen; acetate of copper, sugar or albumen; prussic acid, aqua ammonia; opium, strong decoction of tea, coffee, or nut galls; acetic acid, magnesia or carbonate of potash; ammonia, vinegar or other acids; carbonate of lead, sulphate of magnesia; iodine, starch; iodide of mercury, albumen; chloride of barium, sulphate of soda, or magnesia; nitric acid, magnesia, or carbonate of potash; oxalic acid, chalk or magnesia; sulphuric acid, magnesia or chalk; tartaric acid, chalk, potash, or magnesia.

Another point which should be kept constantly in mind in practice is, a catalogue of the most common medicines which are usually prescribed together, but which are chemically incompatible, and should never be given in combination. When two incompatible medicines are given, a decomposition and re-union take place,—the two are changed in composition, and two new ones, different from either of the first, are formed. The new compounds may be inert or poisonous, and at least, they are certain to produce a different effect from what was expected. In this way the physician deceives himself; he is puzzled at seeing an old familiar medicine produce new and strange effects,—and is led to condemn new ones, because he has not allowed them to have their legitimate action. It has often been the case, that a bungling unchemical composition has been prescribed by the scientific physician, or mixed together by the nostrum vender, which nevertheless produced wonderful medicinal effects. This was owing to the formation of some new substance, of the presence of which they were ignorant, and which might have been prescribed with less trouble and propriety.

A few of the incompatible medicines are the following: acids with alkalies generally; alkaline carbonates with strong acids; tincture of muriate of iron with gum arabic; cyanate of potash with salts of iron; alum with chalk and mercury; tartarized antimony with vegetable astringents; nitrate of silver with vegetable astringents and spring water; uva ursi with carbonate of soda; nut galls with acetate of lead, opium, or ointment of nitrate of mercury; extract of logwood with acetate of lead; calomel with lead, carbonate of soda, soap, magnesia, seidlitz powders, and sulphate of potash; yellow cinchona with camomile, cloves, catechu, cascarilla, and nearly all vegetable bitters; mucilage with acetate of lead; rhubarb with sulphate of iron and magnesia; valerian with sulphate of iron and nitrate of silver; kino with acetate of lead; Fowler's solution of arsenite of potash with sulphuric acid, most salts and hydro-sulphates; lime water with alkaline carbonates, and most vegetable bitters and astringents; opium with sulphate of zinc or copper; antinomial wine with tincture of blood root, laudanum or other astringents; almost all salts with milk, spring water, mucilage, gelatine, and astringents; quinine with nitric acid.

Many other medicines which are incompatible, are often prescribed in practice, even among those who make no small pretensions to science, and profess to be entirely read up in all branches of the profession. But whatever opinions they may entertain of their own skill, the combination of a poor chemist and a good practitioner is quite as incompatible as any in the materia medica. This might have been doubted a few years ago; but since the blaze of light from modern chemistry has shone upon our former system of therapeutics, it has swept it away like vapor before the sunbeam. And in fact, with our present ignorance of practical chemistry, we are left almost without a substitute. While some practitioners are laboring to deduce the entire practice from chemical principles, and thereby constantly making dangerous experiments or failures, others discard all new improvements, and plod their way by the dim and doubtful light of past experience. So that the aggregate of empiricism in medical practice, is perhaps as great as at almost any past time. This is no fault of chemistry; it is only for want of systematic knowledge, that men refuse to make this science the basis of therapeutics, or fail in its practical application when they do so. It is true that chemical facts have come upon us so fast that we have had no time to generalize, classify and apply them in practice. No book exclusively on chemical therapeutics, chemical pathology, or chemical practice, has yet been written. Chemical facts which are applicable in practice, are scattered through large volumes, and concealed by abstruse language, so that much labor is required to collect and arrange them.

Among medical men, chemistry is the last branch read or discussed; among medical students, it is the last branch taken up, and the one least studied. And a student could pass an examination before any corps of professors, provided his only deficiency should be that of giving all the incompatible prescriptions in the materia medica, sooner than one who should fail to tell whether there are six or thirteen layers to be raised in the operation for hernia; although by his incompatible prescriptions he might daily endanger the lives of his patients, while he might not have occasion to operate for hernia during his lifetime.

Simon's Chemistry of Man, Liebig's Animal and Vegetable Chemistry, Liebig on the Motion of the Juices in Animal Bodies, and on Endosmosis and Exosmosis, Bird on the Urine, Elliottson on Poisons, Garrodson's Lectures on Chemical Pathology, and Pereira on the Chemistry of Food and Diet, are books which should be read by every intelligent physician.

*Rochester, June* 3, 1850.

